# 

**Published:** 1912-04-13

**Authors:** 


### Medical Members of the Advisory Insurance Committee.

The following are the sixteen qualified medical practitioners with personal experience of general practice who have been appointed members of the Advisory Committee :—

(*a*) Nominated by the British Medical Association (13) : John Adams, M.B., C.M.; R. M. Beaton, M.B., C.M.; T. M. Carter, M.D.; J. S. Darling, M.D., M.Ch.; S. Hodgson, M.D.; J. A. Macdonald, LL.D., M.D.; J. Munro Moir, M.D., J.P.; J. Neal; E. O. Price, M.D., C.M.; D. F. Todd; E. B. Turner, F.R.C.S.; T. Jenner Verrall; A. H. Williams, M.D., C.M.

(*b*) Nominated by the Association of Registered Medical Women (3) : Miss M. H. F. Ivens, M.S.; Miss C. E. Long, M.D. Brux.; Miss A. M. Watson.

The following are the seventeen medical men selected by the Commissioners : Christopher Addison, M.D., M.P., Secretary of Board of Intermediate Medical Studies, University of London; Sir T. Clifford Allbutt, M.D., K.C.B., Regius Professor of Physic, University of Cambridge; C. J. Bond, F.R.C.S., Senior Honorary Surgeon, Leicester Infirmary; Sir R. J. Collie, M.D., J.P., Medical Examiner, London County Council; Sir Frederick S. Eve, F.R.C.S., Surgeon, London Hospital; Adam Fulton, M.B., B.Ch., Surgeon, South Yorks, Notts, and Derbyshire Coalfield; R. McKenzie Johnston, M.D., C.M., Consulting Aural Surgeon, Royal Infirmary, Edinburgh; Herbert Jones, Medical Officer of Health, Herefordshire Sanitary Districts; E. J. Maclean, M.D., Senior Gynæcologist, Cardiff Infirmary; H. H. Mills, M.D., Physician in General Practice; G. Reid, M.D., Medical Officer of Health, Staffordshire; J. Robertson, M.D., Medical Officer of Health, Birmingham; Lauriston E. Shaw, M.D., Physician, Guy's Hospital; Prof. R. Stockman, M.D., Professor of Materia Medica and Therapeutics, University of Glasgow; W. E. Thomas, M.D., C.M., Surgeon, Pentre and Tynybedw Collieries and Pentre Engineering Works; Norman Walker, M.D., C.M., Physician, Diseases of Skin, Royal Infirmary, Edinburgh; Prof. G. Sims Woodhead, M.D., Professor of Pathology, Cambridge University.

Among the other persons selected by the Commissioners not mentioned on page 29 we may note : J. P. Gilmour, Pharmaceutical Standing Committee on National Health Insurance; W. J. U. Woolcock, Pharmaceutical Standing Committee on National Health Insurance.

### OUR BUREAU OF INFORMATION.

### The Burden of the Unfilled Beds.

A large proportion of charitable institutions are managed on the basis of part payment from patients. The rent is perhaps guaranteed, or subscribers have been found to build up a fund which supplements the income derived from the insufficient contributions of those for whose benefit the home is conducted. We have received an advance copy of the report of Mrs. Barnett's Convalescent Home at Erskine House, Hampstead, which illustrates this excellent system. The total ordinary expenditure for 1911 amounted to £809. Of this sum rather more than half is met by patients' payments, which amounted to £413. The remainder is contributed by annual subscriptions and donations, by King Edward's Hospital Fund for London, by the Hospital Sunday Fund, by the Guild of Compassion, and by Toynbee Hall, in connection with which the home was established. Erskine House receives most of its patients from the London Hospital, whose Samaritan Fund pays at the rate of 10s. 6d. a week for each, and keeps the beds nearly always full. But in homes of this nature it is found in practice that financial ease depends on a continuous supply of patients all the year round. It is quite true that there is a loss on every one person received, and yet the proposition that numbers are a gain is not so unreasonable as it may appear. For the expenses of rent, taxes, water, gas, coal, domestic upkeep, salaries and wages are continuous, and show no diminution when beds are empty. To have the home working at high pressure with fourteen beds full, and £7 7s. a week regularly coming in, is to be relieved of all anxiety about maintenance, which in this particular home, where the housekeeping is admirable, averages about £6 a week.

We have gone into these figures because we have noted that many small homes are in continual difficulties on account of the intermittent character of their activities. In the height of summer or at holiday seasons such homes are overcrowded. At other times, if unconnected with a general hospital, they are often half empty, the exchequer suffers, and, what is far more important, numerous sufferers who would eagerly take advantage of their benefits if they knew about them are being miserably housed in tenements and cheap lodgings for want of proper information, are imperfectly fed, and a prey to a thousand anxieties.

Our Brotherhood of Social Service aims at introducing a new link between the little homes and the patients who are ready and waiting to take advantage of them. All over the country workers in the social field are seeking out the neglected and forlorn who want this kind of help. As they approach us with their wants and problems we will do our best to put them in touch with the vacant beds waiting to be filled.

A registration fee of five shillings entitles the little home to be placed on our annually published List of Approved Institutions for continual reference, and whenever the home is in receipt of grants from public bodies no further guarantee of its good management is required. In the case of privately managed homes we are compelled to ask for two references or a personal recommendation from a member of the Brotherhood. The amount of available accommodation, the terms under which patients are received, and the advantages offered will be made known in these columns for the benefit of workers, in two successive issues, and once a quarter a list will be inserted of homes recently added.

### Needs and Helpers.

Anxious: Address wanted.—Send stamped and addressed envelope with coupon and we will look up the address for you and post it on.

Miss Milkins: Institution wanted for girl orphan, deaf as result of scarlatina. Bright and very intelligent.—You do not mention the age. The Royal Asylum for the Deaf and Dumb Poor, Margate, receives children between the ages of seven and ten, and gives apprentice fees on leaving. Admission is by election in June and December, or by presentation. Write to the Secretary, and let us know if the child is too old for this Home. Perhaps our readers can help.

Dr. D. R., Bristol: Hospitals in Bahia Blanca.—We are making inquiries, and will let you have full particulars in next week's issue.

Madame V. M.—Please note Rule 5. We shall be happy to make your request known in these columns when that is complied with.

Home for Mild Mental Case.—Will "A B C" communicate with Miss Layton, Cotswold, Ilfracombe; also with Sister Arnold, 3 Milton Road, West Hartlepool?

Home for Girls with Chronic Hip Disease.—Will F. M. D. communicate with Miss Layton, Cotswold, Ilfracombe? Write also to Erskine House, Hampstead, where such cases are sometimes received for lengthened periods.

Sister E. M.: Can any of our readers give assistance in the following case, which comes to us very strongly recommended?—A widow, aged thirty-two, with two young children (provided for at school) is now under treatment at the Royal National Hospital at Ventnor. Her time expires on May 3, and she is not yet able to undertake any but the lightest kind of work. Both lungs are healing, but she needs good food and care, with freedom from anxiety, for some months to come. Is there any free home which would receive her? She is a trained social worker, and owes her present breakdown to work carried out under unsatisfactory conditions in a London rescue home last summer.

Readers of The Hospital are invited to bring these columns under the notice of their friends who are engaged in philanthropic work of any description. We want the co-operation of district nurses, district visitors, and health visitors. We want the clergy to send us on particulars of cases which need institution treatment. All who are trying to help their neighbours are faced with the problem of fitting the remedy to the need. It is this problem we aim at solving.

### Rules for Correspondents.

Everyone who uses or wishes to help our Bureau of Information must be kind enough to observe the following rules:—

(1) Postal replies cannot be given. Exception will only be made in very special cases at the Editor's discretion.

(2) Queries or answers relating to different cases must be written, or preferably typed, on separate sheets of paper, and the writing must never be crossed.

(3) Questions and replies received not later than Wednesday will, as far as possible, be dealt with in our issue of the following Saturday week.

(4) Letters must have attached the name and address of the sender, with a pseudonym for publication if preferred.

(5) Applications referring to non-dependents where the need relates to business or employment must be accompanied by a postal order for 5s., when the requirement shall have publicity in these columns.

(6) All applicants for insertion in our list of Approved Homes must send a registration fee of 5s. The omission of this leads to delay and gives avoidable trouble.

(7) *All communications* for this department *must be accompanied by the coupon* to be found at the bottom of the third page of the cover on the inside, and should be addressed to the Editor of the Bureau of Information, c/o The Hospital, 29 Southampton Street, Strand, London, W.C.

### Appointments.

The vacancy for an anæsthetist at St. Thomas' Hospital has been filled by the election of E. V. Dunkley, M.D.

The vacant appointment of surgeon with charge of out-patients at the Great Northern Central Hospital, Holloway Road, has been filled by the election of Mr. Gordon Taylor, B.S., F.R.C.S.

Dr. T. J. Horder has been appointed Assistant Physician to St. Bartholomew's Hospital, at which institution he held for several years the post of Medical Registrar. Dr. Horder is also Physician to the Cancer Hospital and to the Great Northern Hospital. He qualified in 1896, and is M.D. London and F.R.C.P. Dr. Horder is a well-known and prolific writer upon medical topics.

### STOREROOM NECESSITIES.

Such apparently simple necessities as dust-cover sheets and chamois dusters require as much care in choosing as more complicated institutional appliances. Moore, of Lauderdale Buildings, Aldersgate, London, is offering special dust-cover sheets, ten feet long, at 3s. 2d., and chamois dusters at 2s. 9d. per dozen, together with house flannel and swabs at special prices. These should certainly be tried by all institutional managers.

### Florence Nightingale Memorial.

The following contributions have been received by Mr. G. Q. Roberts: The schoolmasters, mistresses, pupil teachers, elder children, and infants of the Army Schools, Aldershot Command, per Major J. H. Howell, Senior Inspector of Army Schools, Aldershot, £14 10s.; Lord Glenconner, £10 10s.; Miss M. A. Webster, £5 13s. 6d.; Lady Lindsay, £5; Major H. N. Schofield, V.C., £5; Mrs. J. W. Cursiter's collection, £3 4s.; Sister Curties, £3 3s.; Northern Cyclist Battalion, Newcastle, £2 6s. 6d.; Noel Trotter, Esq., £2 2s.; Elliot F. Barker, Esq., £1 1s.; Mrs. Douglas McLean, £1 1s.; Major Granville Feilden, £1 1s.; Robert S. Herries, Esq., £1 1s.; T. J. U. Robins, Esq., £1 1s.; A. H. Hobhouse, Esq., £1; 13th Company, R.A.M.C., 13s.; "C" Company, R.A.M.C. Depôt, Aldershot, 10s.

### Gifts and Bequests.

The Royal Cornwall Infirmary has received a bequest amounting to over £6,500 under the will of the late Alderman Dorrington, of Truro. This sum is to be devoted to the establishment of a Convalescent Home for female patients at Perranporth.

The Dowager Lady Quilter has given £500 to the East Suffolk and Ipswich Hospital. This donation is given in accordance with a wish expressed by the late Sir Cuthbert Quilter, Bart. The money will be very welcome to the Board, as further additions to the hospital are about to be started.

Mr. James Cook, of Weston-super-Mare, has bequeathed £1,000 to the Dunster Cottage Hospital, and the residue of his estate, subject to certain other bequests, will be divided equally between the Bridgwater Hospital, the Taunton and Somerset Hospital, and the Royal West of England Sanatorium, Weston-super-Mare, upon trust, of which the income is to be devoted to the general purposes of these institutions.

Mr. Richard Bayly, of Brixton, Devon, has left £1,000 to the South Devon and East Cornwall Hospital, Plymouth, and £500 to the Didworthy Sanatorium for Consumptives.

Mrs. Sarah Lietch has left £1,000 to the National Hospital for the Paralysed and Epileptic, Queen's Square, W.

Mr. Frank Riley-Smith, of Tadcaster, has bequeathed stock to the value of over £2,000 to the Leeds Infirmary and a similar amount to the York County Hospital, and to the Bury and West Suffolk Hospital he has left £500.

Mr. Wm. Henry Alger, of Plymouth, has left £100 to the South Devon and East Cornwall Hospital.

Mrs. Emily Howard, of Salisbury, has bequeathed £1,000 to the West London Hospital and the same amount to the East London Hospital for Children. One third of the residue of the estate is left to the Royal Hospital for Incurables at Putney.

The Dundee Royal Infirmary has received intimations of (1) a legacy of £500 (free of duty) to the Infirmary under the will of the late Mr. John Christie, wholesale grocer, Dundee; and (2) a bequest of one-third of the residue of the estate of the late Miss Catherine McNaughton Campbell, Highfield, Blairgowrie (another third goes to Dr. Barnardo's Homes, and the remaining third to Quarriers' Homes, Bridge of Weir). The amount of the fund to be divided is between £2,800 and £2,900, subject to Government duty and expenses.

### Books Received.

Henry Frowde: "Bronchial Asthma," by J. B. Berkart, M.D.

Chas. Knight and Co., Ltd.: "Everybody's Guide to the National Insurance Act, 1911," by Thomas Smith.

Longmans, Green and Co.: "Manual of Surgical Treatment," by Cheyne and Burchard.

"The Prescriber" Offices: "The Prescriber." Editor, T. Stephenson, F.R.S.E.

Smith, Elder and Co.: "Practical Zoology," by Marshall and Hurst.

John Ouseley, Ltd.: "The Blood," by A. Béchamp.

F. Davidson and Co. (Proprietors): "Sight Testing for the General Practitioner," by F. Davidson.

H. J. Glaisher: "The Feast of Herbs."

Columbia University Press: "Scientific Features of Modern Medicine," by Lee.

Truth Publishing Co.: "*Truth* Cautionary List for 1912."

Methuen and Co., Ltd.: "Round the Red Lamp," by A. Conan Doyle.

The St. Clement's Press, Ltd.: "The Key to Perfect Health," by Arthur Hallam.

James Nisbet and Co., Ltd.: "Tuberculin in the Diagnosis and Treatment of Tuberculosis," by W. Camac Wilkinson; "Nisbet's Medical Directory, 1912."

Scientific Press, Ltd.: "Day Nurseries and their Management," by Bernard E. Myers, M.D.

Meister Lucius and Brüning, Ltd.: "The Tuberculins and their Employment in Diagnosis and Treatment of Tuberculosis."

Effingham Wilson & Co.: "A Guide to the National Insurance Act," by H. Wippell Gadd.

Animal Defence and Anti-Vivisection Society: "Evidence," by Miss Lind-af-Hageby.

Baillière, Tindall and Cox: "Treatment of Tuberculosis and Lupus with Allyl Sulphide," by W. C. Minchin. "Lessons on Massage," by Mrs. M. Palmer.

Cassell and Co.: British Red Cross Society. No. 1, "First-Aid Manual," by James Cantlie, M.A., M.B.

### PRACTICAL BOOKS FOR INSTITUTION WORKERS.

| | s. | d. |
|---|---|---|
| Burdett's Hospitals and Charities. | 10 | 6 |
| Furnishing and Appliances of a Cottage Hospital. | 0 | 6 |
| Modern Nursing in Hospital and Home. | 2 | 6 |
| The Consumptive Working Man. What can Sanatoria do for Him? | 10 | 6 |
| National Insurance and the Voluntary Hospitals. A Perfected System or Cruelty to the Insured. | 0 | 2 |
| The National Insurance Act and Hospital Benefit: How to Prepare for a Perfected Hospital and Health Service for Great Britain. What the Amending Act should contain. | 0 | 1 |
| National Insurance as it affects the Voluntary Hospitals. | 0 | 1 |

Ledgers, Account Books, &c., of all kinds for large or small Institutions.

THE SCIENTIFIC PRESS, LTD., 28-9 Southampton Street, Strand, W.C.

